# Direct and Competitive Optical Grating Immunosensors for Determination of *Fusarium* Mycotoxin Zearalenone

**DOI:** 10.3390/toxins13010043

**Published:** 2021-01-08

**Authors:** Inna Székács, Nóra Adányi, István Szendrő, András Székács

**Affiliations:** 1 Nanobiosensorics Group, Institute for Technical Physics and Material Science, Centre for Energy Research, Konkoly-Thege M. út 29-33, H-1121 Budapest, Hungary; szekacs@mfa.kfki.hu; 2 Food Science Research Institute, National Agricultural Research and Innovation Centre, Herman O. út 15, H-1022 Budapest, Hungary; adanyi.nora@eki.naik.hu; 3 MicroVacuum Ltd., Kerékgyártó u. 10, H-1147 Budapest, Hungary; istvan.szendro@microvacuum.com; 4 Agro-Environmental Research Institute, National Research and Innovation Centre, Herman Ottó út 15, H-1022 Budapest, Hungary

**Keywords:** mycotoxin, zearalenone, immunosensor, optical waveguide lightmode spectroscopy, label-free detection

## Abstract

Novel optical waveguide lightmode spectroscopy (OWLS)-based immunosensor formats were developed for label-free detection of *Fusarium* mycotoxin zearalenone (ZON). To achieve low limits of detection (LODs), both immobilised antibody-based (direct) and immobilised antigen-based (competitive) assay setups were applied. Immunoreagents were immobilised on epoxy-, amino-, and carboxyl-functionalised sensor surfaces, and by optimising the immobilisation methods, standard sigmoid curves were obtained in both sensor formats. An outstanding LOD of 0.002 pg/mL was obtained for ZON in the competitive immunosensor setup with a dynamic detection range between 0.01 and 1 pg/mL ZON concentrations, depending on the covalent immobilisation method applied. This corresponds to a five orders of magnitude improvement in detectability of ZON relative to the previously developed enzyme-linked immonosorbent assay (ELISA) method. The selectivity of the immunosensor for ZON was demonstrated with structural analogues (α-zearalenol, α-zearalanol, and β-zearalanol) and structurally unrelated mycotoxins. The method was found to be applicable in maize extract using acetonitrile as the organic solvent, upon a dilution rate of 1:10,000 in buffer. Thus, the OWLS immunosensor method developed appears to be suitable for the quantitative determination of ZON in aqueous medium. The new technique can widen the range of sensoric detection methods of ZON for surveys in food and environmental safety assessment.

## 1. Introduction

Zearalenone (ZON) is a mycotoxin produced by several *Fusarium* species, most frequently by *F. graminearum*, and is commonly found in maize and also in wheat, barley, sorghum, and rye throughout various countries of the world, causing substantial human exposure [1]. ZON and its metabolites have oestrogenic activity in several species [2,3,4,5] accompanied by hepatotoxicity, haematotoxicity, immunotoxicity, and genotoxicity [6,7,8]. No uniform regulations have been imposed for this toxin in different countries. Tolerance levels in grains and grain products have been set in several countries at a concentration range of 20 to 1000 µg/kg [9], e.g., 20 to 200 µg/kg in unprocessed and processed cereal products in the EU [10]. Data evaluation on the most sensitive animal species—swine—and comparing with humans, a tolerable daily intake for ZON has been set as 0.25 µg/kg body weight [11,12].

Common analytical methods for identifying and quantifying mycotoxins include thin-layer chromatography (TLC) [13,14] or high-pressure TLC [15], laser fluorimetry [16], gas chromatography (GC) [14] often coupled with mass spectrometry (GC-MS) [17,18], high-performance liquid chromatography (HPLC) [19,20,21] with standardised sample preparation [22,23], ultra-performance liquid chromatography (UPLC) [24], and capillary electrophoresis [25,26]. HPLC methods have become the most widespread for mycotoxin analysis. These methods are sensitive and accurate but require extensive sample preparation steps, well-trained personnel, and expensive instrumentation. Therefore, just as for other mycotoxins, and on the basis of the historical radioimmunoassay method [27], immunochemical methods, e.g., enzyme-linked immunosorbent assays (ELISAs), have been developed and utilised for rapid screening of ZON [14,19,28,29,30,31,32,33]. These immunoassays were further amplified with fluorescent quantum dots [34,35,36], magnetic nanoparticles [37], or helical carbon nanotubes [38]. Alternatively, antibodies [24,36,39,40,41,42,43,44] or molecularly imprinted polymers [45,46,47] could be applied for affinity chromatography or pre-column sample purification prior to chromatographic analyses (HPLC, UPLC). Similarly, nanoparticle-assisted lateral flow immunochromatographic strips [48,49] were devised, occasionally with surface-enhanced Raman scattering detection [50]. Recently, micro- and nanoarray immunoassays were reported in microplate-based [51] or microfluidic sensor-based [52] setups. A cut-off level of 100 µg/kg was established (4 min) for ZON and T2 toxin in a gel-based immunoassay [53]. Fluorescence polarisation immunoassays allowed for a detection range for ZON of 150–1000 µg/kg and a limit of detection (LOD) of 137 µg/kg, and required less than 2 min per sample to carry out [54]. A magnetic nanotag-based immunoassay [55] and a multiplexed quantum dot immunochromatographic assay [56] allowed the parallel detection of ZON in the presence of other mycotoxins. Label-free biosensors on the basis of antibodies [57,58,59,60], aptamers [58,61,62,63,64,65,66,67], or molecularly imprinted polymers [68,69,70,71] as recognition elements have also been developed with various signal amplification and detection routes involved, and the range of sensoric detection techniques is expanding [72]. Thus, a surface plasmon resonance (SPR) biosensor has been developed for the simultaneous detection of four mycotoxins, with an LOD below 0.2 ng/mL for ZON [73], a gold nanoparticle-amplified imaging SPR (iSPR) biosensor allowed an LOD for ZON of 59.2 pg/mL in multiplex mycotoxin determination [74], a method of total internal reflection ellipsometry (TIRE) allowed detection of ZON at concentrations as low as 0.1 ng/mL [75], and electrochemical sensors resulted in LODs of 0.15–0.25 pg/mL [42,60]. The immunosensors developed allow rapid quantitative determination of the target compounds in plant samples and in environmental matrices, mainly in ground water.

Immunosensors based on the technique of optical waveguide lightmode spectroscopy (OWLS) have been applied with success to detect different molecules, and gained importance in environmental and food analysis [59,76,77]. In the current study, an OWLS immunosensor has been developed for the determination of ZON in maize samples. Different chemical methods for functionalisation and accordingly for immobilisation were compared regarding analytical sensitivity and sensor stability. Upon optimisation, the novel immunosensor was used for the detection of ZON contamination in maize and the results were compared to ELISA measurements to demonstrate the outstanding applicability of the method in complex food matrices and assumedly, in environmental samples as well.

## 2. Results and Discussion

**Figure 1:**
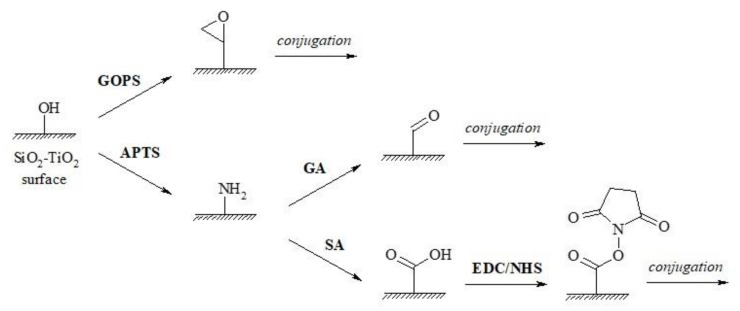
Functionalisation of the sensor surface with epoxy, amino, or carboxyl functional groups. GOPS: γ-glycidoxypropyl-trimethoxysilane; APTS: (3-aminopropyl) triethoxysilane; GA: glutaraldehyde; SA: succinic anhydride; EDC: 1-ethyl-3-(3-dimethylaminopropyl) carbodiimide; NHS: N-hydroxysuccinimide.

OWLS immunosensors were devised both in the direct (immobilised antibody) and competitive (immobilised antigen conjugate)-based formats. Immobilisation of the protein reactants has been carried out by several chemical routes utilising hydroxyl groups on the sensor surface converted into epoxy or amino functionalities, further reacted with appropriate chemical reagents for covalent immobilisation of the protein immunoreagents (Figure 1).

### 2.1. Direct Immunosensor

**Figure 2:**
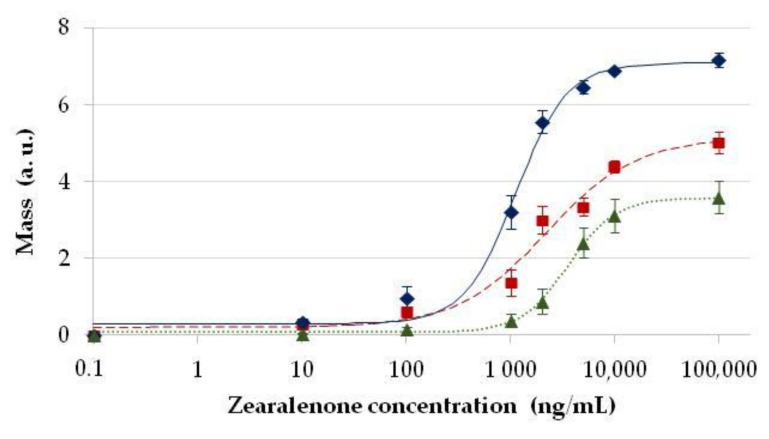
Standard calibration curves for zearalenone (ZON) determination by the direct optical waveguide lightmode spectroscopy (OWLS) immunosensor format. Sensor signals proportional to relative surface mass (ng mm^−2^) on the OWLS sensor, expressed in arbitrary units (a.u.), as a function of concentration of ZON applied in the calibration standard samples in the sensor format with ZON-specific serum immobilised on amino- and epoxy-modified sensor surfaces using (3-aminopropyl) triethoxysilane and glutaraldehyde (APTS/GA) (■, red dashed line), (3-aminopropyl) triethoxysilane, succinic anhydride and 1-ethyl-3-(3-dimethylaminopropyl) carbodiimide with N-hydroxysuccinimide (APTS/SA/EDC-NHS) (♦, blue solid line), and γ-glycidoxypropyl-trimethoxysilane (GOPS) (▲, green dotted line).

Immunoglobulin (IgG) fractions purified from ZON-specific rabbit antisera obtained against a conjugate of ZON to conalbumin (ovotransferrin, CONA) as an immunogen were used in an immobilised antibody-based (direct) immunosensor format. The main characteristics that determined achievable assay signals were the quality and concentration (dilution) of the ZON-specific antibodies. Using a dilution of the IgG purified from the serum of 1:2000 for immobilisation by all three methods, the epoxy-functionalised sensor surface modified with γ-glycidoxypropyl-trimethoxysilane (GOPS), as well as the amino-functionalised sensor surface modified with (3-aminopropyl)triethoxysilane (APTS), and glutaraldehyde (GA) or succinic anhydride with 1-ethyl-3-(3-dimethylaminopropyl)carbodiimide and N-hydroxysuccinimide (SA/EDC-NHS), standard calibration curves were obtained for ZON determination by applying ZON onto the immobilised antibodies on the sensor surface at various concentrations up to 100 μg/mL (Figure 2).

The highest sensor signals were obtained by APTS/GA modification, followed by APTS/SA-EDC/NHS, while immobilisation with GOPS provided the lowest assay signals. Detection sensitivity, characterised with the analyte (ZON) 50% effective concentrations (EC_50_) corresponding to the half-maximal signal level, indicated EC_50_ values of 3.6 ± 0.2, 2.2 ± 0.6, and 1.1 ± 0.1 µg/mL for the GOPS, APTS/GA, and APTS/SA-EDC/NHS modifications, respectively. Signal intensities and statistics indicated that immobilisation on the epoxy-modified surface (GOPS) provided lower binding efficacy and reproducibility than that on amino-modified surfaces (APTS) with homo-bifunctional cross-linking (GA) with further modification to carboxyl groups (SA/EDC-NHS). Nonetheless, the lowest detectable ZON concentrations in these setups were in all three cases above 500 ng/mL, which is not sufficiently sensitive for analysis of real samples.

### 2.2. Competitive Immunosensor

#### 2.2.1. Serum Titration

**Figure 3:**
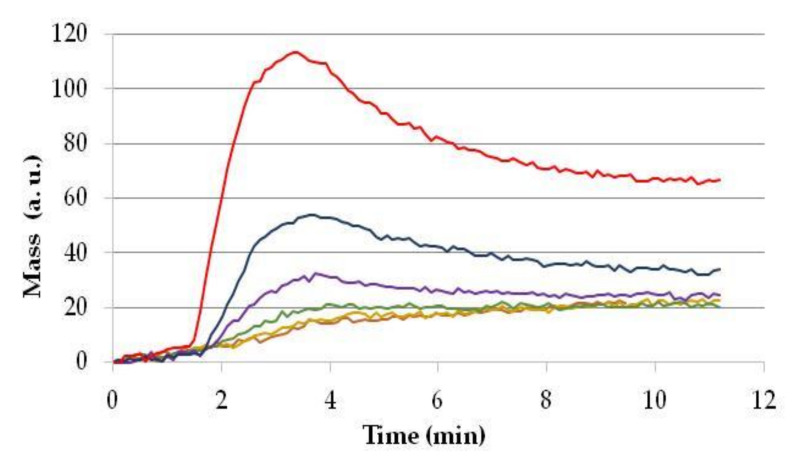
Optimisation of serum dilution by recording sensor responses to polyclonal antiserum at various dilutions using a sensor surface modified with 10 µg/mL of zearalenone conjugate to bovine serum albumin. Serum dilutions at 1:500 (red line), 1:1000 (blue line), 1:2000 (purple line), 1:4000 (green line), 1:8000 (yellow line), and 1:16,000 (brown line).

The polyclonal IgG fraction purified ZON-specific rabbit antisera obtained against ZON-CONA as an immunogen were titrated in the OWLS immunosensor setup using a protein-heterologous conjugate to bovine serum albumin (BSA) at a ZON-BSA concentration of 10 µg/mL as a sensor surface antigen. Purified antisera were injected onto this sensor surface at increasing concentrations (decreasing dilutions) to assess the binding affinity of the antibodies. Typical titration curves are shown in Figure 3, indicating the peak signals obtained in the flow-through system in 3.4 min upon injection, decreasing peak intensities with increasing serum dilution and optimal dilution (the highest dilution still allowing distinguishable signal) at a serum dilution of 1:2000. The use of more concentrated serum for further competitive measurements results in deteriorated method sensitivity, while lower antibody concentrations allow for less stable sensor performance.

The determination of the amount of polyclonal IgG applied is essential in both the direct and indirect (competitive) measurements, particularly in the latter as it is a rather sensitive equilibrium. As seen in Figure 3, when the IgG is applied at small concentrations (high dilutions, e.g., 1:8000 or 1:16,000), antibodies poorly saturate the sensor surface, and small, unstable sensor responses are obtained. On the contrary, in the case of high IgG concentrations (dilutions of 1:500 or 1:1000), although the signal obtained is well measurable (exceeding 100 or 50 arbitrary units, respectively), the surface becomes saturated, and it loses its sensitivity during the measurement of standards and samples. For the measurements, we chose an IgG concentration that is high enough to provide well-measurable signals (at least 20 arbitrary units). On the other hand, the IgG concentration should not be too high, so that the system remains sensitive enough to detect standards containing low amounts of the antigen. Taking the height and shape of the signals into consideration, in the case of competitive measurement of ZON, the dilution of the antibody solution was chosen to be 1:2000.

#### 2.2.2. Competitive Immunosensor Setups with Different Surfaces Modifications

**Figure 4:**
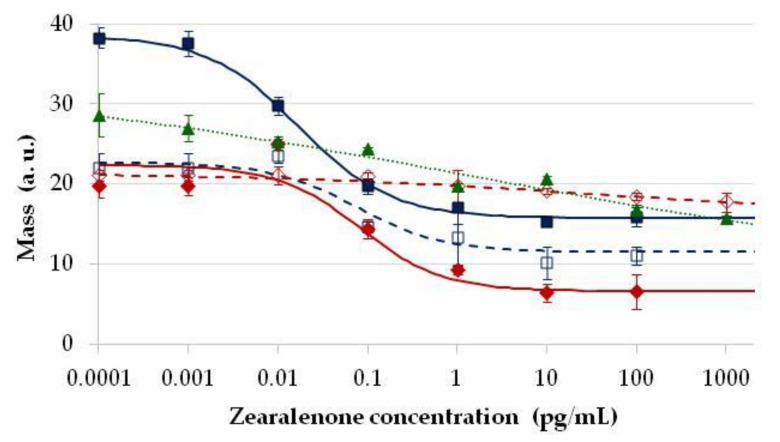
Standard calibration curves for zearalenone (ZON) determination by the competitive optical waveguide lightmode spectroscopy (OWLS) immunosensor format. Sensor signals proportional to relative surface mass (ng mm^−2^) on the OWLS sensor, expressed in arbitrary units, as a function of concentration of ZON applied in the calibration standard samples in the sensor format with ZON conjugate to bovine serum albumin (ZON-BSA) immobilised on amino- and epoxy-modified sensor surfaces with (3-aminopropyl) triethoxysilane and glutaraldehyde (APTS/GA) using 10 µg/mL ZON-BSA (■, blue solid line) and 5 µg/mL ZON-BSA (□, blue dashed line); (3-aminopropyl) triethoxysilane, succinic anhydride and 1-ethyl-3-(3-dimethylaminopropyl) carbodiimide with N-hydroxysuccinimide (APTS/SA/EDC-NHS) using 10 µg/mL ZON-BSA (♦, red solid line), 5 µg/mL ZON-BSA (◊, red dashed line), and γ-glycidoxypropyl-trimethoxysilane (GOPS) (▲, green dotted line).

The protein-heterologous conjugate of ZON (ZON-BSA) was used as a sensor surface antigen at various concentrations between 2 and 20 µg/mL with the above three immobilisation methods. Upon serum titration, surface coating conditions were optimised for immunosensor sensitivity i.e., analytical standard curves were obtained using concentration series of ZON and recording its inhibitory effect on antibody binding to the treated immunosensor surfaces (Figure 4).

The highest and stable sensor signals were obtained when ZON-BSA was at 10 µg/mL concentration, above which further improvement in assay signals could not be obtained, and excess of the antigen even caused less reproducible or deteriorated signals. Similarly to the direct sensor format, immobilisation on the epoxy-modified surface (GOPS) was found to be of limited utility in the competitive sensor format as well. Although addition of ZON resulted in concentration-dependent sensor signals, nonetheless, this means of immobilisation was improper for analytical purposes due to the lack of proper regression possibility with the four-parameter logistic fitting. Immobilisation on amino- (APTS/GA) or carboxyl-modified surfaces (APTS/SA/EDC-NHS) allowed for better quantitative detection possibilities, with dynamic detection ranges of 0.001–1 pg/mL when the antisera were used in 1:2000 dilution. Immobilisation with GA appeared to be applicable at coating levels with ZON-BSA both at 10 and 5 µg/mL, while the SA/EDC-NHS method resulted in a standard sigmoid curve only at a coating level of 10 µg/mL. In these cases, linear detection ranges were found to be similar with good reproducibility. Antisera obtained from different rabbits (under the same immunisation protocol), although showing somewhat different titration characteristics, provided similar results in the competitive formats, indicating that slight differences in serum composition did not have inhibitory activity. The highest sensor signals were obtained here also by APTS/GA modification. Detection sensitivity showed outstandingly low EC_50_ values in the range of 0.017–0.083 pg/mL, corresponding to at least six orders of magnitude improvement in detection range compared to the direct immunosensor. The dynamic detection range of ZON was found in the 0.010–1 pg/mL ZON concentrations, and an LOD for ZON of 0.002 pg/mL was obtained in the APTS/GA modification using the ZON-BSA conjugate as the surface coating antigen at 10 µg/mL. As the competitive immunosensors based on amino- and carboxyl-modified surfaces provided similar ranges of detection, due to the better reproducibility and longer shelf-life of the latter, the sensor setup using 10 µg/mL of ZON-BSA as the surface coating antigen immobilised with the APTS/SA/EDC-NHS method, as well as ZON-specific antibodies at 1:2000 dilution, was chosen to be used for practical purposes.

### 2.3. Immunosensor Specificity

Immunosensor specificity was tested on the optimised immunosensor setup (see above) by measuring EC_50_ values obtained with ZON derivatives and structurally unrelated mycotoxins, and cross-reactivities (CRs), defined as a percentage ratio between the EC_50_ values of ZON and the given compound, were calculated. Among the structurally unrelated compounds tested, aflatoxin B1 and ochratoxin did not cause a decrease in the OWLS sensor signal up to 1000 ng/mL concentration in the diluted standards. Among the compounds tested, only α-zearalenol, α-zearalanol, and β-zearalanol showed significant CRs (Table 1) with ZON in the competitive immunosensor format. These are major reductive metabolites of ZON in mammals, but are also formed to a lesser extent in plants as well [12]; therefore, the potential presence of these metabolites should be also considered upon positive detection of ZON in commodities by the current immunosensor method. These CR values are in good agreement with the corresponding values reported for our ELISA system for ZON [29]; however, the detection sensitivity of the current OWLS immunosensor exceeds that of the ELISA by five orders of magnitude. Such outstanding improvement in the detection range of an OWLS immunosensor compared to the corresponding ELISA has been reported [59,78].

### 2.4. Method Validation in Commodity Matrix

**Figure 5:**
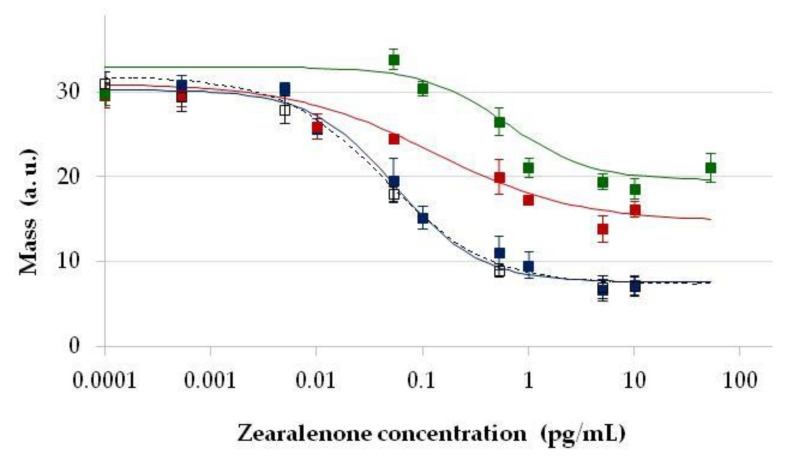
Standard calibration curves for zearalenone (ZON) determination by the competitive optical waveguide lightmode spectroscopy (OWLS) immunosensor format in maize extract at various dilutions. The amino-modified sensor surface with ZON conjugate to bovine serum albumin (ZON-BSA) immobilised at 10 µg/mL on amino-modified sensor surfaces by (3-aminopropyl) triethoxysilane, succinic anhydride and 1-ethyl-3-(3-dimethylaminopropyl) carbodiimide with N-hydroxysuccinimide (APTS/SA/EDC-NHS) using ZON-specific serum at 1:2000 dilution. Maize extracts were applied at dilutions of 1:100 (■, green line), 1:1000 (■, red line), and 1:10,000 (■, blue line); ZON calibration curve in 42 mM 2-amino-2-(hydroxymethyl)-1,3-propanediol (Tris) buffer (pH 7.4) (□, black slashed line).

The optimised competitive immunosensor was applied to determine ZON concentrations in maize commodity. For this purpose, maize samples spiked with ZON at concentration levels of 0–10 µg/kg were extracted in acetonitrile/water (6:4), and were analysed by the competitive OWLS immunosensor and ELISA. These analyses aimed to assess matrix effects by the maize extract on the one hand, and were also targeted to investigate whether the two analytical methods detect the same ZON concentrations, identical to the nominal values, on the other hand. To assess possible matrix effects on immunosensor performance, the aqueous extracts were diluted 1:100 to 1:10,000 in 42 mM 2-amino-2-(hydroxymethyl)-1,3-propanediol (Tris) buffer (pH 7.4). Figure 5 shows analytical standard curves obtained by the optimised competitive immunosensor setup in diluted maize extracts, and demonstrates that matrix effects are diluted out at 1:10,000. ZON concentrations detected by the competitive immunosensor indicated analytical recoveries at initial ZON concentrations between 5 ng/kg and 10 µg/kg, carried out in triplicates, were found to be 84% and 124%, mostly suitable for practical use. It has to be noted, however, that the maximal recovery value fell by 3.3% out of the acceptable recovery range of the European legislation performance criteria for ZON detection set to be 60–120% and 70–120% for ZON concentration at or below 50 µg/kg and above 50 µg/kg, respectively [79].

**Figure 6:**
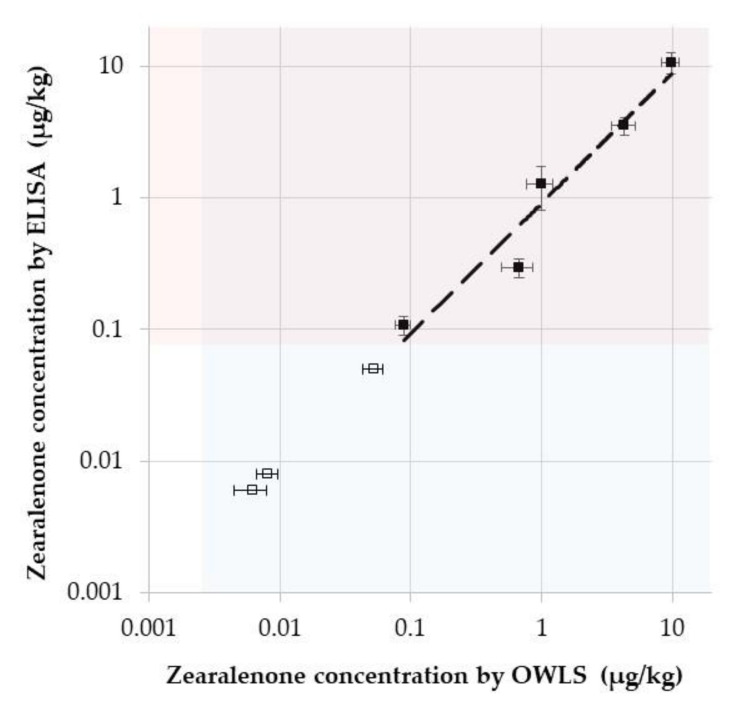
Determination of zearalenone (ZON) content in maize samples by optical waveguide lightmode spectroscopy (OWLS) immunosensor and enzyme-linked immunosorbent assay (ELISA). The sensing range for OWLS (>0.002 µg/kg) and for ELISA (>0.09 µg/kg) are indicated in blue and red, respectively. (Ordinate values for ZON concentrations below 0.09 µg/kg are virtual for visualisation—indicated by hollow rectangles).

ZON concentrations measured in maize extract by OWLS and ELISA methods were compared to each other as shown in Figure 6. Results indicate that concentrations detected by the two methods well correlated with each other in the 0.1–10 µg/kg range (r^2^ = 0.984), and both methods are applicable. However, while the ELISA method required an extract dilution of 1:10 and detected ZON above 0.1 ng/mL, the OWLS immunosensor required an extract dilution of 1:10,000, but detected ZON above 0.01 pg/mL.

## 3. Conclusions

To provide stable immunosensors for the detection of mycotoxin ZON, continuous flow OWLS sensor setups were established. To immobilize protein immunoreagents (ZON-specific antibodies or ZON-BSA conjugate), the immunosensor surface was modified by epoxy, amino, and carboxyl functional groups under laboratory conditions by optimised silanisation protocols. Epoxy functional groups allowed direct immobilisation of the proteins under alkaline conditions (pH = 9.5). Amino functional groups allowed direct immobilisation of the proteins with 2.5% GA, or could be converted to carboxylic acid functional groups by 0.2% SA and conjugate to proteins using a 1:1 mixture of 0.1 M NHS and 0.4 M EDC.

In the direct (immobilised antibody) format, immobilisation on epoxy-modified surfaces (GOPS) provided lower binding efficacy and reproducibility than that on amino- (APTS/GA) or carboxyl-modified surfaces (APTS/SA/EDC-NHS). However, detectable ZON concentrations fell in all three cases above 500 ng/mL, not being sufficient for practical purposes. In the competitive (immobilised antigen) format, immobilisation on epoxy-modified surfaces (GOPS) remained improper for use, not providing sigmoid analyte concentration dependence, but amino- or carboxyl-modified surfaces were found of high utility. Both methods (APTS/GA and APTS/SA/EDC-NHS) resulted in similar analytical detection levels (EC_50_ values in the range of 0.017–0.083 pg/mL) and linear detection ranges. Higher signal levels—therefore, greater signal decreases by inhibition—were achieved with amino-modified surfaces; however, carboxyl-modified surfaces allowed for more stable and reproducible results. The optimised competitive immunosensor using 10 µg/mL of ZON-BSA as the surface coating antigen immobilised by the APTS/SA/EDC-NHS method, as well as ZON-specific antibodies at 1:2000 dilution, was found to show excellent sensitivity and specificity to ZON, allowed an LOD of 0.002 pg/mL, and was found to be applicable to the determination of ZON in maize extracts. Detectable analyte concentrations in assay buffer were found to be five orders of magnitude lower by the immunosensor than by the related ELISA method, which, considering the sample preparation requirements, corresponds to a three orders of magnitude improvement for determination of ZON content in maize commodity. Such unique improvements in the analytical sensitivity of the OWLS technique compared to the corresponding ELISA method have previously been evidenced for the detection of other analytes, including a nearly three orders of magnitude enhancement for the endocrine biomarker protein vitellogenin [77] and a six orders of magnitude improvement for a herbicide active ingredient trifluralin [76]. Moreover, the current OWLS immunosensor represents substantial advancements compared to previous immunosensors for ZON, e.g., based on SPR [71], TIRE [73], and electrochemical detection with antibodies immobilised on gold nanoparticles embedded on multi-walled carbon nanotubes [42], to which the LOD of the current competitive OWLS immunosensor represents 30,000-, 5000-, and 75-fold improvements, respectively.

## 4. Materials and Methods

### 4.1. Reagents and Instrumentation

Chemical reagents, including γ-glycidoxypropyl-trimethoxysilane (GOPS) and (3-aminopropyl) triethoxysilane (APTS), mycotoxin standards, proteins, and biochemicals, were purchased from Sigma-Aldrich Kft. (Budapest, Hungary) unless indicated otherwise. OWLS immunosensor measurements were carried out on an OWLS 210 instrument and BioSense 3.8 software (MicroVacuum Ltd., Budapest, Hungary) using OWLS 2400 sensor chips with optical grating of 2400 lines per mm in the SiO_2_-TiO_2_ waveguide layer (MicroVacuum Ltd., Budapest, Hungary). Immunoassays were carried out in an iEMS MF microplate reader (LabSystems, Helsinki, Finland) using high-capacity 96-well microplates (Nunc, Roskilde, Denmark).

### 4.2. Immunogen and Antibody Production

Since ZON is low-molecular-weight hapten, it is non-immunogenic and should be conjugated to a protein carrier for immunisation. ZON was converted to the corresponding hapten, ZON-6′-carboxymethyloxime, and was conjugated to carrier proteins BSA and CONA by the method from the literature [26]. Polyclonal antibodies directed against ZON were produced in female, 3-month-old New Zealand white rabbits immunised periodically and intradermally with a standard mixture of 25 μg of the ZON-CONA conjugate immunogen per kg body weight and 50 μL of Freund’s complete or incomplete adjuvant. Rabbit immunisation was carried out under the supervision of the Ethics Committee of Research on Animals (Food Science Research Institute, National Agricultural Research and Innovation Centre, Budapest, Hungary) and under the authorisation and inspection by the Government Office for Pest County in Hungary (Official permit for animal testing # PE/EA/45-6/2020, last date of approval: 21 February 2020). Serum from the whole blood obtained was centrifuged at 2400 g for 15 min, and its IgG fraction was purified by sodium-sulphate precipitation [80].

### 4.3. OWLS Immunosensor Measurements

OWLS sensoric determinations were carried out in a flow-through cell of the OWLS 210 instrument. The optical grating of the sensor surface is illuminated with a polarised He-Ne laser light (632.8 nm), and the sensor chip is rotated along its axis in a narrow angle range (±7°). The laser beam is diffracted on the grating, and enters the waveguide at the characteristic incoupling angles, where it propagates by total internal reflection, and is detected by photodiodes at the ends of the waveguide layer. Incoupling of the incident laser beam occurs at two well-defined angles of incidence: one for transverse electric (TE) and one for transverse magnetic (TM) mode. Rotating the cuvette with ±7 degrees, effective refractive indices (NTE and NTM) are monitored, and four characteristic photocurrent peaks (TE and TM peaks on both, positive and negative sides) can be detected at the incoupling angles αTE and αTM. Apparent incoupling angles can be measured with 10^−4^ degree accuracy, and signal resolution relative to the effective refraction index is ΔN~10^−6^. The mass of the deposited material absorbed on the waveguide surface from the continuous-flow medium can be calculated from the effective refractive indices (NTE and NTM), expressed as thickness of the protein layer deposited (nm) or surface coverage (ng cm^−2^). All determinations were carried out at room temperature in a flow-injection analyser system at a flow rate of 200 µl/min and with injection volumes of 200 µL.

#### 4.3.1. Functionalisation of the Sensor Surface

The sensor surface was derivatised by several routes to form reactive functional groups for covalent immobilisation of the protein immunoreagents (ZON-specific antibodies or ZON-BSA conjugate) (Figure 1). Proteins were immobilised to the derivatised sensor chip in a flow-through system using a 42 mM Tris running buffer (pH 7.4). Reactive epoxy groups were formed on the sensor surface by heating the sensor chips to 60 °C in 10% GOPS in toluene for 20 hrs, followed by washing the chips with toluene and further heating to 100 °C for 1 hr. Epoxy functionalised surfaces allowed for direct anchoring of biomolecules carrying amino or hydroxy moieties by nucleophilic addition to them in alkaline medium (pH > 8.5) [81]. Thus, protein immunoreagents were injected onto the epoxylated sensor chips at 1–20 µg/mL concentrations in 0.2 M carbonate buffer at pH 9.5, followed by buffer exchange to 42 mM Tris buffer at pH 7.4, and removal of unbound proteins from the surface by injecting 0.1 M aqueous hydrochloric acid. Amino groups were formed on the surface of the sensor by treating the chips at 75 °C with 10% APTS at pH 3.0 for 4 hrs, followed by washing with distilled water and heat treatment at 95 °C for 6 hrs [81]. The amino functionality was activated with 2.5% aqueous glutaraldehyde (GA), allowing direct additive anchoring biomolecules carrying amino groups. The immobilisation reaction was carried out within the flow-through cuvette, by injecting GA into the flowing distilled water medium, followed by medium exchange to Tris buffer (42 mM, pH 7.4), subsequent injection of the proteins at 1–20 µg/mL concentration, and elution of the unbound reagent fraction by the injection of 0.1 M aqueous hydrochloric acid. Alternatively, amino groups were modified to carboxyl groups by derivatisation with succinic anhydride (SA), and were utilised for covalent attachment of biomolecules by the activated ester method using NHS with a dehydrating agent EDC. Carboxylation was carried out in separate vessels with 1% SA in dry ethanol at 25 °C for 1 hr, followed by drying the chips at 90 °C for 15 min. Active ester formation on the chip surface was carried out in a stopped flow mode by injecting a 1:1 solution of 0.2 M EDC and 0.05 M NHS, incubating at room temperature for 10 min, rinsing with Tris buffer (42 mM, pH 7.4), adding the protein-ZON conjugate at 1–20 μg/mL concentration in 10 mM sodium acetate buffer at pH 4.0, and incubating again at room temperature for 10 min. Alternatively, active ester formation could be carried out outside the OWLS 210 instrument, on a Petri dish, under similar reaction conditions. Residual active ester functional groups were finally deactivated by the injection of 1 M ethanolamine at pH 8.5 for 10 min.

#### 4.3.2. Immunosensor Formats

Non-competitive and competitive detection formats were applied for developing OWLS immunosensors. The first format was based on the immobilisation of 2000-fold diluted polyclonal antibodies. Such immobilised antibodies capture their analyte (the antigen or similar immunoreactive compounds) from the sample; therefore, this format is often also termed a direct format, and the amount of antigen bound to the immobilised antibodies is proportional to the quantity of the antigen in the standard solutions. In the second format, 10 µg/mL of ZON-BSA conjugate was bound to the solid surface, then standards or samples were mixed in a 1:1 ratio with solutions containing known amounts of antibodies, and the mixture upon a short incubation was injected into the system. The amount of antibodies bound to the immobilised conjugates is inversely proportional to the quantity of the antigen in the standard solutions.

### 4.4. Sample Preparation

Ground maize samples were spiked with ZON at the concentration range of 5 ng/kg to 10 µg/kg (5, 10, 50, 100, and 500 ng/kg and 1, 5, and 10 µg/kg). One gram aliquots of the spiked samples were extracted with 10 mL of acetonitrile/water (6:4) as a solvent. The samples were stirred for 10 min and centrifuged on ultrafiltration membranes with a 100,000 nominal molecular weight limit at 5000 rpm for 10 min, and the filtrate was collected for OWLS and ELISA measurements. Upon sample preparation, all samples were stored at 4 °C until measurement and were diluted with 42 mM Tris buffer (pH 7.4) to the appropriate rate prior to analysis.

### 4.5. Determination of ZON by ELISA Method

To confirm the utility of the immunosensor, ZON content was also determined in a corresponding competitive ELISA system [29]. ELISA plates were coated with 5 µg/mL ZON-BSA conjugate, and inhibition of binding of the polyclonal antibody by ZON was measured using a commercial horseradish peroxidase labelled second (anti-rabbit IgG) antibody and a colorimetric immunoassay signal measured at 450 nm.

### 4.6. Data Analysis and Statistics

All determinations, except for the real time recordings in direct sensor titration experiments (Figure 3), were performed at least in triplicates, and error bars on the graphs represent the standard deviation (SD) of the replicates for each datum point. SDs were calculated as the square root of variance of the deviation of each datum point relative to the mean. Sigmoid calibration curves were obtained by logistic mathematical fitting using the Rodbard equation [82], which were also used for determination of the IC_50_ values. LOD values were defined as an analyte concentration corresponding to a signal that differs from the background level by 3 SDs of the background.

## Figures and Tables

**Table 1:** Percentage of cross-reactivity (CR%) of the competitive OWLS immunosensor and the corresponding ELISA method [29] with zearalenone and its derivatives.

Compound	OWLS Sensor		ELISA	
	IC_50_ (pg/mL)	CR% ^1^	IC_50_ (ng/mL)	CR% ^1^
zearalenone	14.3	100	14.1	100
α-zearalenol	56.5	25.2	50.1	28.2
α-zearalanol	111.0	12.8	199.5	7.1
β-zearalanol	526.5	2.7	1259.0	1.1

^1^ Cross-reactivity defined as the percentage ratio of the 50% inhibitory concentration (IC_50_) values of zearalenone and of the given derivative.

## Data Availability

The data presented in this study are available on request from the corresponding author. The data are not publicly available due to privacy reasons.
